# 3D Muscle Volume and 3D Fat Fraction After Successful and Failed Arthroscopic Rotator Cuff Repair at 5-Year Follow-up

**DOI:** 10.1177/03635465241299795

**Published:** 2025-03-01

**Authors:** Philipp Kriechling, Jethin Joshy, Stefan Klotz, Georg C. Feuerriegel, Philipp Fürnstahl, Reto Sutter, Mazda Farshad, Karl Wieser

**Affiliations:** † Department of Orthopaedics, Balgrist University Hospital, University of Zurich, Zurich, Switzerland; ‡ Department of Radiology, Balgrist University Hospital, University of Zurich, Zurich, Switzerland; § Research in Orthopaedic Computer Science, Balgrist University Hospital, University of Zurich, Zurich, Switzerland; Investigation performed at Balgrist University Hospital, Forchstrasse, Zürich, Switzerland

**Keywords:** clinical outcome, midterm outcome, rotator cuff repair, segmentation, 3-dimensional fatty infiltration, volume measurement

## Abstract

**Background:**

The results of rotator cuff (RC) repair surgery can be influenced by the presence of muscle atrophy and fatty infiltration. Three-dimensional (3D) quantitative data regarding those degenerative muscle changes after successful or failed RC repair are rare in the current literature.

**Hypothesis/Purpose:**

The purpose of this study was to analyze muscle volume and fatty infiltration of the supraspinatus muscle after successful and failed arthroscopic RC tendon repair, with a minimum follow-up of 5 years. It was hypothesized that RC muscle volume and fatty infiltration would improve after successful repair and only to a limited extent after failed repair.

**Study Design:**

Cohort study; Level of evidence 2.

**Methods:**

A total of 115 patients (mean age, 59 ± 8 years; 33% women) with RC repair for full-thickness supraspinatus tendon tear were prospectively followed at 3 and 12 months. Of them, 18 patients with unsuccessful RC repairs were matched to 21 patients with successful repairs and reevaluated at a minimum follow-up of 60 months. All patients underwent quantitative 2-point Dixon magnetic resonance imaging at all time points to evaluate full 3D muscle volume and 3D fatty infiltration. The clinical examination included the full Constant-Murley score (CS) and subjective shoulder value.

**Results:**

The relative changes in supraspinatus muscle volume were statistically significant between the 2 groups over time (*P* < .01). Successful repairs showed a mean volume increase of 18% (*P* < .001) and 23% (*P* < .001) from preoperatively and the 3-month follow-up, respectively, and failed repairs were remodeled by 3% (*P* = .586) and 12% (*P* = .001), respectively. However, a direct comparison of the volumes revealed comparable results at the latest follow-up with 43 cm^3^ (95% CI, 38-47 cm^3^) and 40 cm^3^ (95% CI, 33-46 cm^3^) for successful and failed repairs (*P* = .494), respectively. The supraspinatus 3D fatty infiltration also showed lower fat content for the successful repair preoperatively (6.9% [95% CI, 4.7%-9.2%] vs 9.1% [95% CI, 7.2%-11.1%]; *P* < .01), at 3 months (7.9% [95% CI, 5.5%-10.4%] vs 12.8% [95% CI, 9.1%-16.5%]; *P* < .01), at 12 months (7.5% [95% CI, 4.8%-10.1%] vs 11.6% [95% CI, 9.4%-13.8%]; *P* < .01), and at 60 months (7.4% [95% CI, 4.7%-10.2%] vs 15.5% [95% CI, 11%-20%]; *P* < .01) postoperatively. Fatty infiltration remained unchanged between preoperatively and from 3-month follow-up in the successful group. However, it increased by 70% (*P* < .01) from preoperative and by 21% (*P* = .065) from 3-months follow-up in the failed group. The clinical outcome was similar for successful and failed repairs with an absolute CS of 81 ± 6 versus 72 ± 15 (*P* = .069) and a relative CS of 94% ± 7% versus 85% ± 17% (*P* = .078).

**Conclusion:**

Successful RC repair was associated with relevant improvement of supraspinatus muscle mass and an unchanged fatty infiltration at a midterm follow-up of 5 years. However, failed repairs achieved only mild improvement of supraspinatus muscle volume and showed deterioration of fatty infiltration.

Rotator cuff (RC) tendon tears are known to be among the most common orthopaedic injuries and their occurrence has been shown to increase as the population ages.^29^ Symptomatic RC tears are characterized by pain and limited function. Because of the high prevalence, RC repair is one of the most commonly performed orthopaedic soft tissue procedures, leading to restoration of shoulder function and alleviation of pain.^5,39^ However, high retear rates of 10% to 40% or even more have been reported in the literature and discussed to theoretically limit the potential success of the procedure.^5,10,39,42,48,49^ A large number of studies have identified preoperative muscle atrophy and fatty infiltration to be associated with higher failure rates and inferior clinical outcomes.^18,20,26,35,39^ Muscle volume atrophy was originally described by Thomazeau et al^54^ and Zanetti et al^62^ utilizing qualitative systems to describe atrophy. Assessment of fatty infiltration was described by Goutallier et al^19^ using a 5-stage computed tomography–based classification system and further modified by Fuchs et al^12^ into a 3-stage magnetic resonance imaging (MRI)–based qualitative classification system. Both classifications were based on the most lateral parasagittal view showing the full scapula Y (connection between the scapula, the base of the coracoid, and the base of the acromion).^12,19^

Further studies focused on the potential changes postoperatively looking at muscle volume and fatty infiltration over time.^¶^ This large body of evidence is hampered by some important issues. First, most studies used a 5-stage^19^ or 3-stage^12^ qualitative assessment, which is less precise compared with quantitative methods. This limitation has recently been overcome by the introduction of a proton chemical-shift imaging technique that acquires water-only and fat-only images from a dual-echo acquisition quantitative MRI such as Dixon or IDEAL,^8,41,47^ which allows for an exact description of the amount of fat rather than a rater-dependent classification. This leads to the second shortcoming that most studies utilized a single parasagittal slice to evaluate the muscle volume and fatty infiltration. Subsequently, a few recent studies^9,37,38,41,55,57,59,60^ have started to assess the 3-dimensional (3D) volume of the RC focusing initially on preoperative data and short-term outcomes. Usually, only 1 postoperative time point was analyzed in most studies, leading to the third limitation of postoperative imaging as a reference point after RC repair, which has been popularized by Jo and Shin^26^ The authors recommended using postoperative imaging as a reference for further evaluation of volume and fatty infiltration to better account for the restoration of the potentially retracted muscle-tendon unit.^26^ Subsequently, evaluation of muscle volume and fatty infiltration could start at that postoperative time point in addition to assessment from the preoperative time point.^3,16,21,26,34,45,51^

In summary, the optimized setup would include a prospectively conducted study with quantitative 3D assessment of the supraspinatus muscle for muscle volume and fatty infiltration over a satisfactory period with multiple follow-up time points—including an additional postoperative time point as reference analysis. However, the evidence in the literature is limited. Therefore, the study aimed to analyze 3D muscle volume and 3D fatty infiltration at a minimum follow-up of 5 years. It was hypothesized that RC muscle volume and fatty infiltration would improve after successful repair and only to a limited extent after failed repair.

## Methods

### Ethical Approval

Ethical approval for the study was obtained from the local ethics committee, and all patients gave informed consent before enrollment in the study, ensuring compliance with ethical guidelines. The study was conducted following the Declaration of Helsiniki.^58^

### Patients

This study investigated patients of a previously described prospectively enrolled cohort at a midterm follow-up of at least 5 years using the same methods.^57^ The original cohort included 115 patients with RC repair of at least a large tear of the supraspinatus muscle who underwent MRI preoperatively, 3 months postoperatively, and 12 months postoperatively. The exclusion criteria of the original study were previous surgery, osteoarthritis or inflammatory arthritis of the investigated shoulder, the use of oral steroids, or disagreement to participate in the study. The surgical technique was described previously.

Of those 115 patients,^57^ patients with repair failure classified as Sugaya IV or V^53^ at the 12-month follow-up were matched to patients with successful repairs using preoperative tear size (Patte and Cofield classifications),^4,46^ age, and sex as matching criteria. At the midterm follow-up, an attempt was made to retain the same patients; however, because of a 15% (6 patients) dropout rate, additional participants were selected from the original cohort to ensure the same total number.

### Primary Outcome

The primary outcome measures were 3D supraspinatus muscle volume and 3D supraspinatus muscle fatty infiltration at all time points comparing failed repairs with successful repairs.

### Secondary Outcome

The secondary outcome measures included clinical outcomes and complications.

### Radiographic Evaluation

All patients were evaluated preoperatively and at 3 months, 12 months, and a minimum of 60 months postoperatively using a 1.5-T or 3-T MRI scanner (Siemens 1.5T Magnetom Sola and 3T Magnetom Prisma) with 2-point or 6-point Dixon sequences,^32^ which are chemical-shift based imaging techniques that acquire water-only and fat-only images from a multiecho acquisition, allowing quantification of the intramuscular fat content. Moreover, the 3-month follow-up imaging was considered as the second reference time point for evaluation in addition to the preoperative time point. All images were analyzed by 1 fellowship-trained radiologist (G.C.F.). The 2-dimensional (2D) radiographic evaluation included initial classification of the tear size according to Cofield^4^ and Patte,^46^ as well as tear location that included anterior part, posterior part, or both for supraspinatus assessment and superior part, inferior part, or both for infraspinatus and subscapularis evaluation. Retears after surgical intervention were defined as Sugaya IV/V,^53^ and successful repairs defined as Sugaya I and II.^53^ Also, 2D quantitative fat fraction analysis was accomplished by selecting the most lateral slice displaying the scapular Y shape and placing thereon fat signal fraction maps.^14,28,62^ Further, the tangent sign was used using the lateral y-view to evaluate muscle hypotrophy.^62^

Moreover, 3D muscle volume and 3D fatty infiltration were measured after segmentation of the Dixon MRIs, which all included the complete supraspinatus muscle. As previously described,^57^ the MRI data were obtained from the Image Archiving and Communication System (Merlin PACS Version 7.1, Phoenix-PACS GmbH) and stored as Digital Imaging and Communications in Medicine files. Segmentation was achieved by manually outlining the supraspinatus muscle in 3–mm thick planes on Eco0 sequences with MeVisLab (Version 3.4.3; MeVis Medical Solutions AG). The resulting voxel data were exported into the CASPA software (Version 5; Computer Assisted Surgery Planning Application, Balgrist CARD AG) to calculate the precise volume. Assessment of 3D fatty infiltration was achieved by exporting the previously defined region of interest into ITK-Snap (Version 4.0.2; Penn Image Computing and Science Laboratory, University of Pennsylvania) and assessment on Dixon sequences. Due to changes in the MRI devices and slight differences in MR protocols at the latest follow-up time point, a correction factor of 2.0 was applied to determine fatty infiltration. This correction factor was calculated by scanning 15% of the cohort twice also including the primary MRI device and protocols from the first study. The 3D evaluation of the supraspinatus volume and fatty infiltration was performed independently by 2 physicians with a special interest in muscle segmentation (J.J. and S.K.) and blinded to the clinical data.

### Clinical Outcome

The clinical evaluation comprised the Constant-Murley score,^6^ which included abduction force measurement using a hand-held dynamometer, the subjective shoulder value (SSV)^17^ and the QuickDASH.^1^ All clinical examinations were performed by 1 examiner with a special interest in shoulder surgery (J.J.) or 1 study nurse.

### Statistical Analysis

The statistical analysis was conducted using SPSS statistical software Version 24.0 (IBM Corp). Continuous data were presented as the mean and standard deviation, and absolute data as the number (%). Normal distribution was assessed using the Shapiro-Wilk test and visual inspection. As most data were distributed nonnormally, the Mann-Whitney *U* test and the Wilcoxon signed rank test were used to compare the groups as appropriate. Post hoc power analysis for the setting of independent and matched groups was used to calculate the power of the clinical results and 3D measurements at the latest follow-up (G*Power Version 3.1.9.6, Heinrich-Heine-Universität Düsseldorf)). Spearman rank correlation was utilized to assess an association between muscle volume and fatty infiltration. Interrater reliability for assessment of 3D volume and 3D fatty infiltration was calculated using intraclass correlation coefficient (ICC) using a 2-way model to provide the results of “agreement” and “consistency” calculation. Significance was set at *P* < .05.

## Results

**Figure 1.**
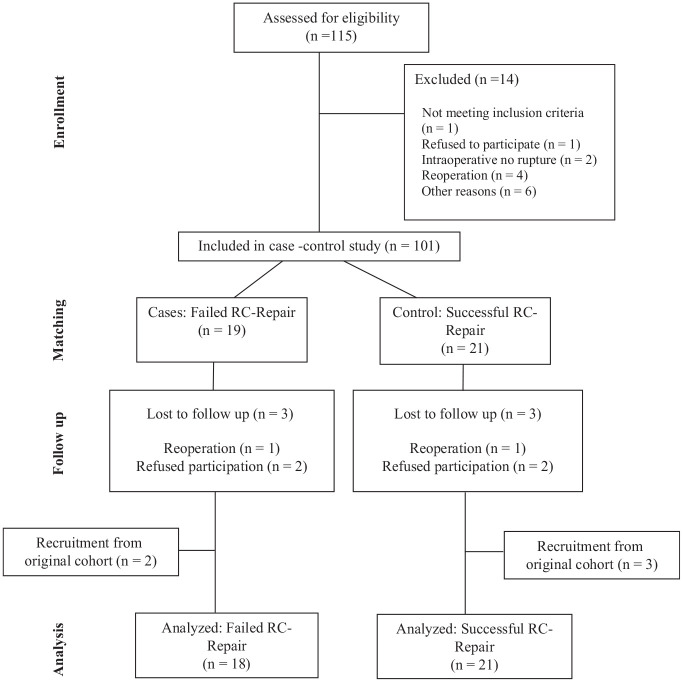
Flowchart detailing the inclusion and exclusion process. RC, rotator cuff.

A total of 39 patients were included, with a mean age of 62 ± 6 years, and 21% were women (see Figure 1 for flowchart and Table 1 for patient characteristics). The mean follow-up duration was 71 ± 10 months.

**Table 1:** Intergroup Comparison of Patients Characteristics, Clinical Data, Rotator Cuff Tear Patterns (Cofield and Patte Classifications), and Muscle Degeneration According to Goutallier Preoperatively^*a*^

		Intact		Failure		
	Total	n^*b*^	%	n^*b*^	%	*P*
Patients	39	21		18		
Sex						.70^*d*^
Female	8	5	24	3	17	
Male	31	16	76	15	83	
Side						.40^*e*^
Right	18	11	48	7	61	
Left	21	10	52	11	39	
Dominance						.91^*e*^
Right	37	20	95	17	94	
Left	2	1	5	1	6	
Age,^*c*^ y	62 ± 6	61 ± 6		63 ± 6		.28^*f*^
BMI^*c*^	27 ± 4	27 ± 4		27 ± 4		.63^*f*^
Cofield						.17^*e*^
Medium		15	72	8	44	
Large		3	14	7	39	
Massive		3	14	3	17	
Goutallier SSP						.07^*e*^
Grade 0		10	26	3	8	
Grade 1		10	26	11	28	
Grade 2		1	2	4	10	
Goutallier ISP						.39^*e*^
Grade 0		13	33	8	21	
Grade 1		6	15	9	23	
Grade 2		2	5	1	3	
Goutallier SSC						0.04^*e*^
Grade 0		19	49	10	26	
Grade 1		2	5	6	15	
Grade 2		0	0	2	5	
Patte SSP						.47^*e*^
Grade 1		7	33	5	28	
Grade 2		13	62	10	55	
Grade 3		1	5	3	17	
Positive tangent sign		1	5	3	17	.32^*e*^
Occurrence						≥.999^*d*^
Acute		4	19	4	22	
Chronic		17	81	14	78	
Tear pattern						
Involving ISP		11	28	12	31	.37^*e*^
Involving SSC		15	40	13	34	.85^*e*^
Involving both		6	29	9	50	.17^*e*^
Additional procedures						
Biceps tenotomy		18	86	15	72	.84^*e*^
Biceps tenodesis		1	5	0	0	.34^*e*^
Subacromial decompression		19	91	10	56	.03^*d*^
Acromioplasty		14	67	7	39	.83^*e*^
ACJ resection		1	5	1	5	.91^*e*^

a ACJ, acromioclavicular joint; BMI, body mass index; ISP, infraspinatus; SSC, subscapularis; SSP, supraspinatus.

b Absolute number.

c Mean ± SD.

d Fisher exact test.

e Pearson chi-square test.

f Independent-Samples *t* test.

### Muscle Volume

**Figure 2.**
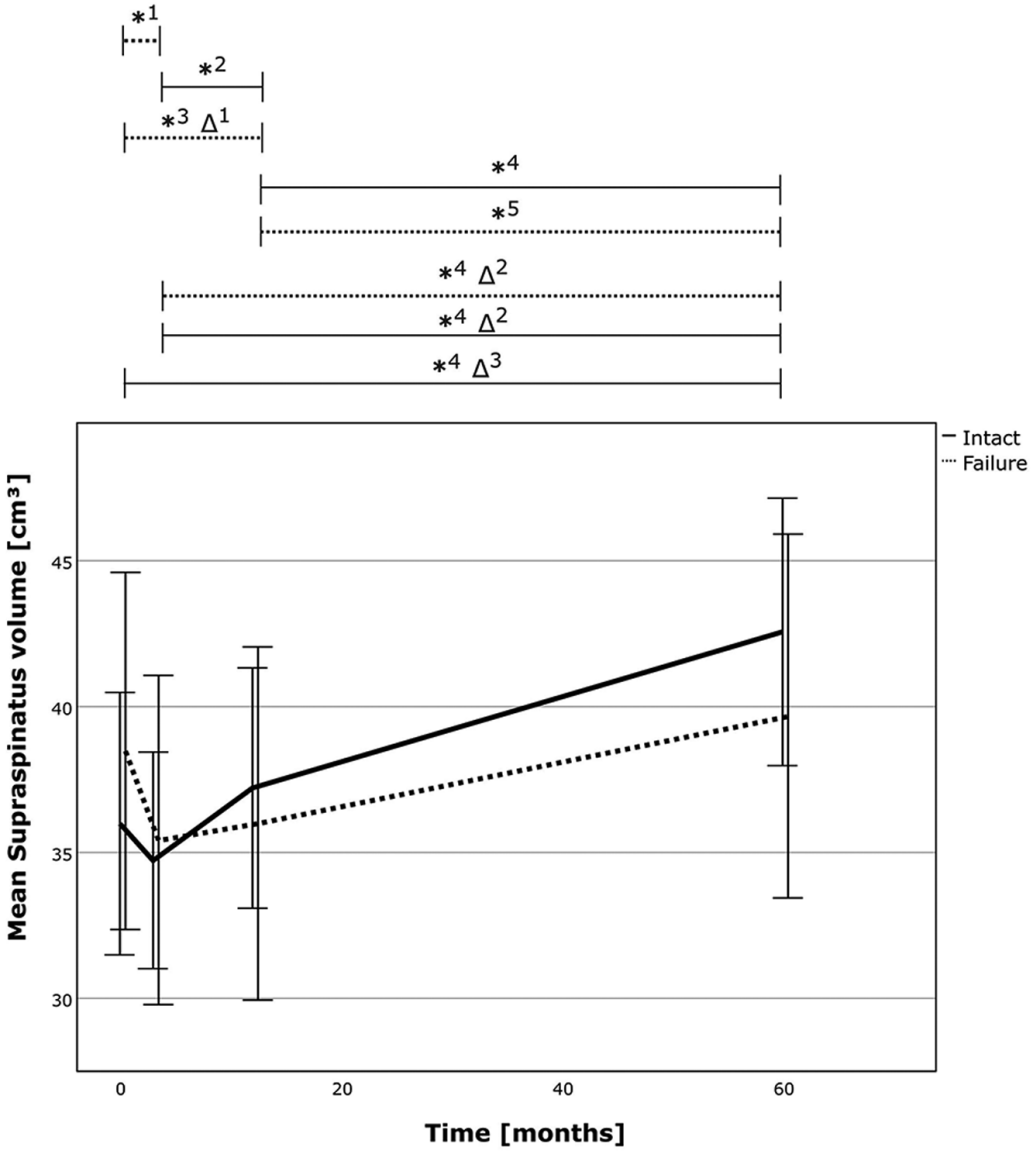
The 3D muscle volume of the supraspinatus over time for the successful RC repair group (solid line) and the failed repair group (dotted line). The asterisk (*) marks statistically significant intragroup development between the 2 time points, and the delta sign (Δ) marks statistically significant relative improvements between time points between the 2 groups. *1, *P* = .004; *2, *P* = .006; *3, *P* = .048; *4, *P*≤ .001; *5, *P =* .002; Δ1, *P* = .015; Δ2, *P* = .022; Δ3, *P* = .006. RC, rotator cuff; 3D, 3-dimensional.

The supraspinatus muscle volume increased significantly in both groups. The increase in muscle volume reached 18% (*P* < .001) and 23% (*P* < .001) in the successful repair group and 3% (*P* = .586) and 12% (*P* = .001) in the failed repair group from preoperative and 3 months postoperatively to the final follow-up, respectively. The relative (delta) volume increase between the groups was more pronounced in the successful RC repair group (*P* = .006). Further, the muscle volume also increased in due course from 12 months to 60 months postoperatively in both groups. However, despite the larger increase in muscle volume after successful repairs and the larger delta increase compared with failed repairs, direct comparison between the groups at the final follow-up was not statistically significant (Table 2, Figure 2, and Appendix Tables A1-A2 available in the online version of this article). Further analysis revealed a power of 0.117 and 0.323 for comparison of muscle volume between the 2 groups at the latest follow-up for independent and matched group calculation, respectively.

**Table 2:** Supraspinatus Muscle Volume and Fat Fraction Preoperatively and 3, 12, and 60 months Postoperatively^*a*^

	Intact (n = 21)						Failure (n = 18)						
	Mean	SD	95% CI	Median	Max	Min	Mean	SD	95% CI	Median	Max	Min	*P* ^*b*^
SSP volume, cm^3^													
Preop	35.99	9.92	31.47-40.50	35.91	55.64	16.99	38.48	12.28	32.37-44.58	35.33	60.38	22.03	.666
3 mo postop	34.72	7.95	31-38.44	35.43	46.92	20.66	35.41	11.35	29.76-41.05	33.05	56.64	19.74	.919
12 mo postop	37.21	9.05	33.09-41.33	38.21	52.62	18.60	35.99	12.14	29.95-42.02	34.12	58.17	17.67	.587
60 mo^*c*^ postop	42.56	10.03	38-47.13	44.72	55.21	25.70	39.66	12.53	33.43-45.89	35.82	58.11	22.20	.494
SSP 2D fat fraction, %													
Preop	6.7	4.5	4.6-8.7	6	16	2	9.2	5.2	6.6-11.7	8	20	3	.112
3 mo postop	6.8	3.6	5.1-8.6	6	16	3	11.6	6.4	8.3-14.9	11	25	2	.012
12 mo postop	6.4	4.7	4.3-8.5	6	25	2	12.5	6.1	9.5-15.5	12	25	5	<.001
60 mo^*c*^ postop	6.6	7	3.4-9.8	5.2	35.1	2	17.7	11.8	11-23.6	13	38	4	<.001
SSP 3D fat fraction, %													
Preop	6.9	4.8	4.7-9.2	5.6	24.8	3.5	9.1	3.8	7.2-11.1	8.3	17.6	4.8	.007
3 mo postop	7.9	5.1	5.5-10.4	6.2	26.1	4.1	12.8	7	9.1-16.5	11.5	31.4	5.6	.003
12 mo postop	7.5	5.6	4.8-10.1	5.7	29.9	3.7	11.6	4.4	9.4-13.8	12.1	21.8	5	<.001
60 mo^*c*^ postop	7.4	6.1	4.7-10.2	6	32.2	2.9	15.5	9.1	11-20	13.5	35.1	6.1	<.001

a Max, maximum; min, minimum; postop, postoperatively; preop, preoperatively; SSP, supraspinatus; 3D, *3-dimensional; 2D, 2-dimensional*.

b Mann-Whitney *U* test.

c At least 60 months postoperatively.

The ICC between the 2 readers (J.J., S.K.) was 0.877 and 0.966 for agreement and consistency, respectively.

### Fatty Infiltration

**Figure 3.**
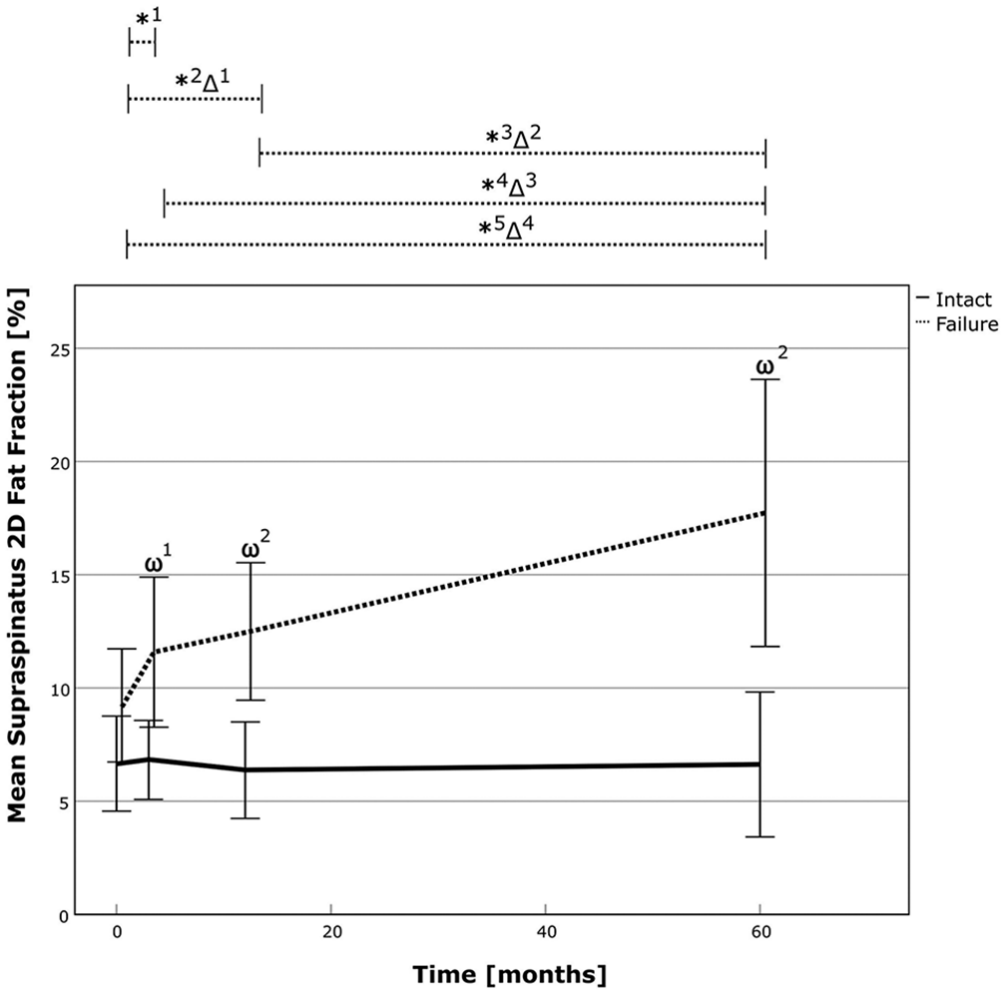
The 2D fat fraction of the supraspinatus over time for the successful RC repair group (solid line) and the failed repair group (dotted line). The asterix (*) marks statistically significant intragroup development between the 2 time points, and the delta sign (Δ) marks statistically significant relative improvements between time points between the 2 groups. The omega sign (ω) highlights a significant difference between the groups at 1 particular time point. *1, *P* = .041; *2, *P* = .032; *3, *P* = .012; *4, *P* = .04; *5, *P* < .001; Δ1, *P* = .028; Δ2, *P* = .018; Δ3, *P* = .044; Δ4, *P* < .001; ω1, *P* = .012; ω2, *P*≤ .001. RC, rotator cuff; 2D, 2-dimensional.

Analysis of 2D fat fraction revealed a steady state in successful repairs and deterioration in failed repairs. It remained unchanged from 6.7% preoperatively or 6.8% at 3 months postoperatively to 6.6% at latest follow-up in the successful repair group and increased from 9.2% preoperatively or 11.6% at 3 months postoperatively to 17.7% in the failure group. Analysis of relative increase between both groups showed a more pronounced amount of fatty infiltration following failed repair (*P* < .01). Over the course of time, fatty infiltration increased by 92% (*P* < .01) and 53% (*P* = .04), from preoperatively and 3 months postoperatively, respectively, in the failed RC repair group (Table 2, Figure 3, and Appendix Tables A1-A2, available in the online version of this article). Further analysis revealed a power of 0.927 and 0.999 for comparison of 2D fatty infiltration between the 2 groups at the latest follow-up for independent and matched group calculation, respectively.

**Figure 4.**
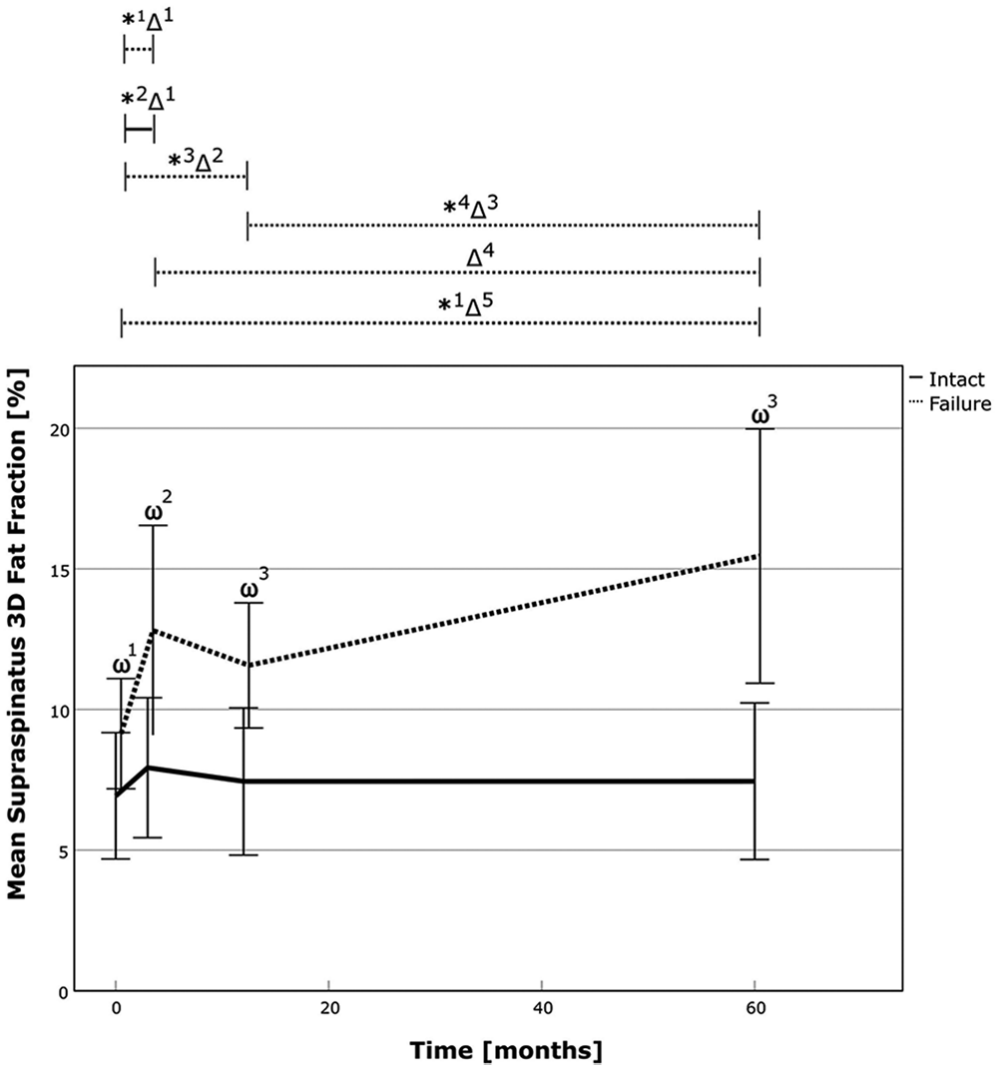
The 3D fat fraction of the supraspinatus over time for the successful RC repair group (solid line) and the failed repair group (dotted line). The asterix (*) marks statistically significant intragroup development between the 2 time points, and the delta sign (Δ) marks statistically significant relative improvements in time points between the 2 groups. The omega sign (ω) highlights a significant difference between the groups at 1 particular time point. *1, P ≤ .001; *2, *P* = .003; *3, *P* = .002; *4, *P* = .009; Δ1, *P* = .046; Δ2, *P* = .01; Δ3, *P* = .026; Δ4, *P* = .024; Δ5, *P*≤ .001; ω1, *P* = .007; ω2, *P* = .003; ω3, *P*≤ .001. RC, rotator cuff; 3D, 3-dimensional.

Analysis of 3D fat fraction revealed no change in successful repairs and deterioration in failed repairs. Comparisons showed better results for successful repair at 3 months (7.9% vs 12.8%; *P* = .003), 12 months (7.5% vs 11.6%; *P* < .001), and 60 months (7.4% vs 15.5%; *P* < .001) postoperatively. In the failed repair group, fatty infiltration increased by 70% (*P* < .01) compared with preoperatively and by 21% (*P* = .065) compared with the 3-month follow-up. (Table 2, Figure 4, and Appendix Tables A1-A2 [available online]). Further analysis revealed a power of 0.827 and 0.999 for comparison of 3D fatty infiltration between the 2 groups at the latest follow-up for independent and matched group calculation, respectively.

The ICC between the 2 readers was 0.734 and 0.893 for agreement and consistency, respectively.

**Figure 5.**
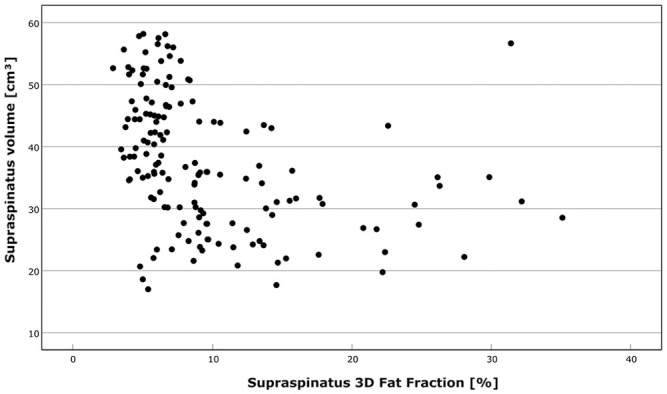
The relationship between the muscle volume and the fat fraction.

Supraspinatus volume and fat fraction were negatively correlated, with a Spearman correlation coefficient of −0.480 and a Pearson correlation coefficient of −0.367 (Figure 5).

### Clinical Analysis

**Figure 6.**
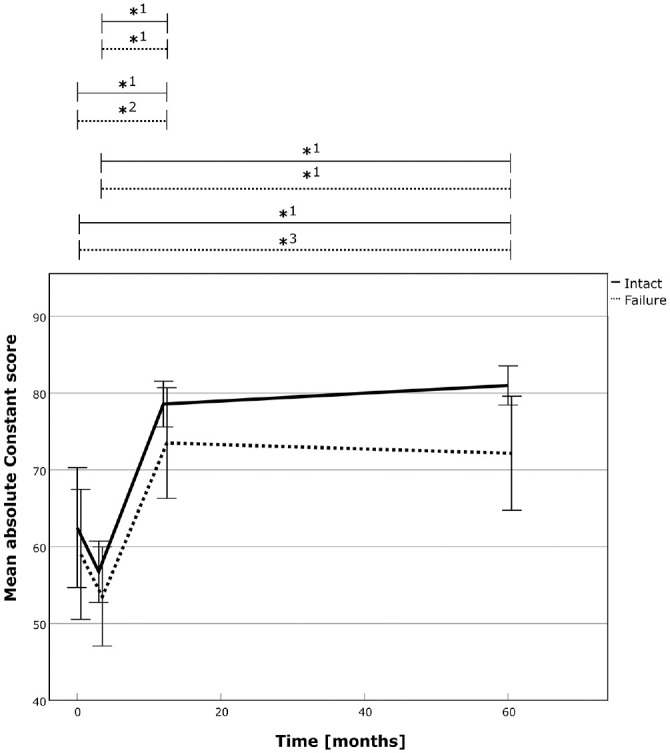
The absolute CS over time for the successful RC repair group (solid line) and the failed repair group (dotted line). The asterix (*) marks statistically significant intragroup development between the 2 time points. *1, *P*≤ .001; *2, *P* = .003; *3, *P* = .013. CS, Constant score; RC, rotator cuff.

**Figure 7.**
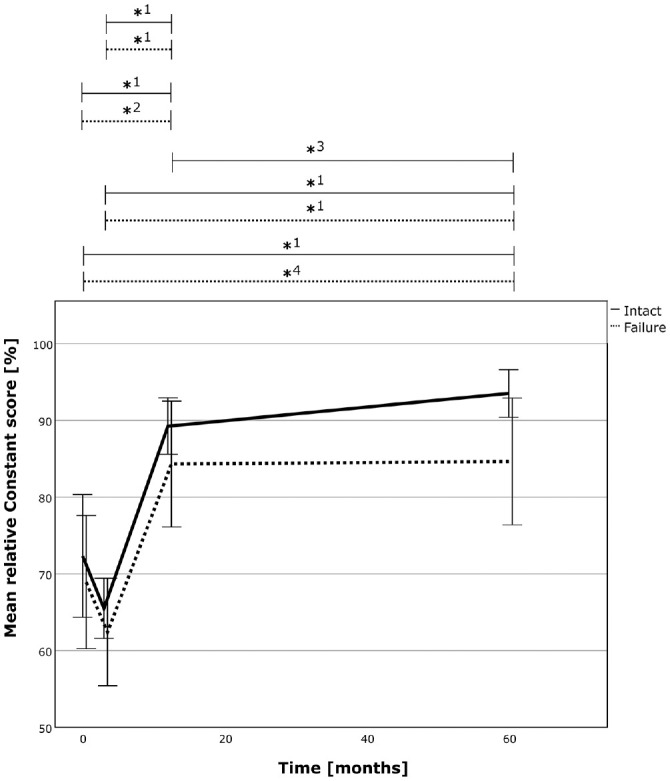
The relative CS over time for the successful RC repair group (solid line) and the failed repair group (dotted line). The asterix (*) marks statistically significant intragroup development between the 2 time points. *1, *P*≤ .001; *2, *P* = .004; *3, *P* = .014; *4, *P* = .003. CS, Constant score; RC, rotator cuff.

**Figure 8.**
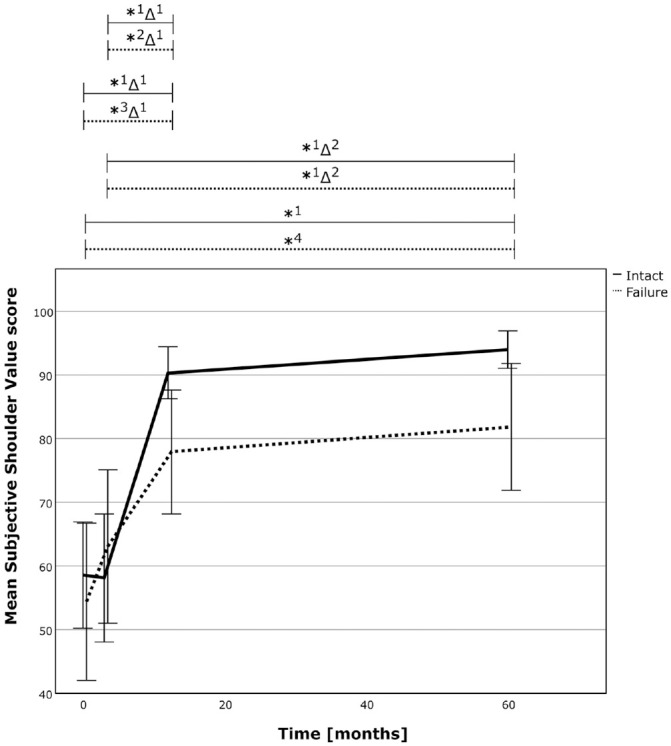
The SSV over time for the successful RC repair group (solid line) and the failed repair group (dotted line). The asterix (*) marks statistically significant intragroup development between the 2 time points, and the delta sign (Δ) marks statistically significant relative improvements between time points between the 2 groups. *1, *P*≤ .001; *2, *P* = .003; *3, *P* = .010; *4, *P* = .007; Δ1, *P* = .005; Δ2, *P* = .008. RC, rotator cuff; SSV, subjective shoulder value.

Analysis of patient-reported outcomes revealed a stable result over the analyzed period, with significant improvements from pre- to postoperatively. The successful repair group achieved a similar result compared with the failure group, with an absolute Constant-Murley score (CSa) of 81 ± 6 (intact) versus 72 ± 15 (failure) (*P* = .069) and a relative Constant-Murley score (CSr) of 94% ± 7% (intact) versus 85% ± 17% (failure) (*P* = .078). The SSV was also similar compared with the repair group (94% ± 7% vs 82 ± 20%; *P* = .140). The results are presented in detail in Table 3, Appendix Tables A3 and A4 (available online), and Figures 6 to 8. The rerupture analysis is shown in Appendix Tables A5 and A6 (available online). Further analysis revealed a power of 0.654 (matched, 0.979), 0.545 (matched, 0.955), and 0.671 (matched, 0.981) for comparison of the CSa, relative CSr, and SSV between the 2 groups at the latest follow-up.

**Table 3:** Absolute and Relative Constant Scores and Subjective Shoulder Value Preoperatively and, 3-, 12-, and 60-month Postoperatively^*a*^

	Intact (n = 21)						Failure (n = 18)						
	Mean	SD	95% CI	Median	Max	Min	Mean	SD	95% CI	Median	Max	Min	*P* ^*b*^
Absolute CS													
Preop	62.48	17.17	54.66-70.29	69	83	25	59	16.98	50.56-67.44	62.50	78	20	.349
3 mo postop	56.76	8.79	52.76-60.76	57	79	41	53.50	13	47.03-59.97	58.50	70	29	.945
12 mo postop	78.57	6.55	75.59-81.55	79	87	65	73.50	14.52	66.28-80.72	78	86	33	.460
60 mo^*c*^ postop	81	5.61	78.45-83.55	81	93	68	72.17	15.01	64.70-79.63	76.50	88	35	.069
Relative CS, %													
Preop	72.31	17.54	64.33-80.30	80.19	95	33.34	68.90	17.47	60.22-77.59	71.40	87.80	30	.477
3 mo postop	65.49	8.63	61.56-69.42	64	87.50	51	62.39	14.13	55.36-69.42	66.50	78	33	.967
12 mo postop	89.23	8.09	85.54-92.91	91.40	99.25	70.60	84.31	16.55	76.08-92.54	89.70	98.75	37	.626
60 mo^*c*^ postop	93.52	6.82	90.42-96.62	94.38	106.40	79	84.64	16.57	76.40-92.88	90.88	101.57	41	.078
SSV, %													
Preop	58.57	18.38	50.20-66.94	60	100	30	54.41	24.04	42.05-66.77	55	100	10	.622
3 mo postop	58.14	22.01	48.12-68.16	60	100	20	63.06	24.14	51.05-75.06	57.50	100	20	.549
12 mo postop	90.29	9.01	86.18-94.39	90	100	70.00	77.94	19.59	68.20-87.68	82.50	100	40	.078
60 mo^*c*^ postop	93.98	6.54	91-96.95	95	100	80	81.83	20.13	71.82-91.84	90	100	40	.140
QuickDASH													
60 mo postop^*c*^	10.28	10.32	5.58-14.97	6.80	36.40	0	17.06	15.20	9.50-24.62	14.75	61.40	0	.140

a CS, Constant score; Max, maximum; Min, minimum; postop, postoperatively; preop, preoperatively; SSV, subjective shoulder value.

b Mann-Whitney *U* test.

c At least 60 months postoperatively.

## Discussion

This study represents a comprehensive follow-up to a previous study aiming to reinvigorate longstanding discourse by leveraging recent technological strides utilizing quantitative analysis to thoroughly evaluate 3D supraspinatus muscle volume and 3D fatty infiltration. This investigation spans a midterm follow-up period of at least 5 years, encompassing a comparison between 3 months postoperatively with latest follow-up. The key findings were that the supraspinatus muscle volume increased significantly over time and that the 3D fat fraction remained unchanged in successful repairs which partly confirmed the hypothesis. Interestingly, supraspinatus muscle volume also improved in failed repairs from the 3-month follow-up and from the 12-month follow-up but to a smaller degree. However, no improvement was seen between preoperatively and the latest follow-up, which might be explained by the limited patient number.

Significant technological developments in the assessment of muscle volume have recently been popularized and were the basis for this study. Those new techniques might represent a milestone in the analysis of RC disease, the subsequent pathophysiological changes, and the guidance of optimal treatment strategies. Evaluation of muscle volume was mainly performed in 2 dimensions using the most lateral parasagittal image where the coracoid base, the spina scapulae, and the margo superior meet (“scapula Y”).^54,62^ In recent years, the evaluation of the 3D muscle volume has gained popularity with the development of new muscle segmentation techniques, which were initially applied only to the lateral part of the muscle.^41^ Further improvement of muscle analysis was achieved by evaluation of the complete muscle including also the most medial part, which is usually not included in a standard MRI of the shoulder. Vidt et al^55^ published in 2016 that analysis of muscle volume in a single parasagittal slice does not represent the overall muscle volume correctly. Similar results were shown by Liu et al^37^ and Jo and Shin.^26^

The present study used 3D muscle volume analysis of the complete supraspinatus muscle and showed a significant improvement in muscle volume from preoperatively and 3 months postoperatively to the latest follow-up in successful and failed repairs. Interestingly, patients with failed RC repairs also gained muscle volume over that period of time but to a much smaller degree than patients with successful RC repairs. One possible explanation for the improvement of muscle volume after failed repair might be partial healing of the tendon or even scarring of the tendon at a nonanatomic position. Matsumura et al^38^ utilized 3D volume assessment for different types of RC tears but without any follow-up after treatment. Chung et al^3^ performed a semi-3D analysis by including the scapula-Y slice and 1 or 2 cm more to the medial and lateral sides. Interestingly, the authors also reported improvements in muscle volume, which also progressed at 1 year. Those results are comparable with the initial presentation of this cohort at a 1-year follow-up.^57^ However, comparability between the studies and the presented work is limited.

Because of the limited number of studies using 3D analysis, data need to be compared with data in studies that used the conventional, qualitative single-slice assessment. Those data are very interesting because the results have been contradictory among studies. Some studies showed deterioration of muscle volume after repair^16,18^ and others showed no improvement.^7,9,11,13,21,35^ However, most studies^2,3,15,16,21,25,44,45,57,61^ reported an increase in muscle volume over time, which is in line with the presented data.

Similar to the muscle volume analysis, evaluation techniques for fatty infiltration have improved largely in recent years to overcome the limitations of single-slice qualitative measurements.^12,19^ Even though the interrater reliability is still known to be unsatisfactory, a large number of subsequent studies^39^ utilized those techniques also because of the practical application in the clinical setting. Recently, evaluation quality improved largely through quantitative evaluation of fatty infiltration using new technologies— such as Dixon or IDEAL sequences (proton chemical-shift techniques).^8,41,47^ A further advancement was the evaluation in 3D rather than in only a single slice. However, these new techniques have not been applied to analyze fatty infiltration over time on a larger scale so far. Two applications of quantitative fat analysis were recently published.^9,57^ However, a few authors have utilized those technological advances in preoperative image analysis to predict clinical outcomes.^24,33,38,41,43^ Vidt et al^55^ found that, as for muscle volume analysis, a single slice analysis is not reliable for analyzing fatty infiltration correctly. The presented analysis revealed a stable level of fatty infiltration following successful RC repair and an increase following failed RC repair.

As described above, no study in the literature exists for direct comparison of 3D data of fatty infiltration. The discussion around fatty infiltration is very interesting because some authors strongly believe that no improvement is possible at all.^7,11,13,16^ However, a few studies reported improvements in fat fraction after RC repair,^20,21,25,57,61^ most studies described no change at all, and a few showed deterioration^3,9,15,18-20,22,30,35,43,45^ which is in line with our findings following successful repair.

Apart from the evaluation of muscle volume and fat fraction, the time point of the analysis might also be of importance. Jo and Shin^26^ popularized the definition of a time-zero analysis of RC repairs, which implies defining a reference time point for data analysis shortly after surgery rather than before surgery. This method was already used by Gerber et al^16^ but without specifically describing it as “time-zero”. The time-zero analysis has been supported by many authors so far,^3,16,21,26,34,45,51^ and it seems reasonable that preoperative evaluation of a torn, retracted, and potentially largely deformed muscle belly might lead to erroneous assumptions. However, the exact time point for time-zero analysis still needs to be defined as one could assume that immediate postoperative hematoma or edema might result in misleading calculations. Therefore, the 3-month follow-up mark was used as an additional reference to the preoperative assessment for analysis.

Apart from multiple reference points for analysis, most studies lack sufficient follow-up data after RC repair. Usually, a minimum follow-up of 1 or 2 years is available in the current literature. However, this might be far too short for such a complex procedure because the strenuous rehabilitation program after RC repair is usually rather restricted than progressive with strengthening exercises starting at the earliest time point of 3 months postoperatively. Frequently, the shoulder becomes stiff after arthroscopic surgery in the first few months, which further complicates proper rehabilitation and subsequent gain of muscle volume. The presented analysis accounted for that potential limitation, with a minimum follow-up duration of 5 years.

Interestingly, the clinical outcome was statistically similar between the patients with successful and failed RC repair albeit the successful group had a tendency for better results. This fact has been discussed extensively in the literature with still conflicting opinions on when to perform RC repair.^29^ Similar results were already seen in the previous publication that revealed no statistically significant difference between the groups at the 12-month follow-up.^56^ Of note, the patient cohort has changed slightly since then because of some dropouts; nonetheless, it fits the same criteria as before. In addition, also the relative gain (delta) was similar between the groups with 30%, 29%, and 60% versus 22%, 23%, and 50% improvements for CSa, CSr, and SSV, respectively. Nevertheless, the successful RC repair group had a tendency for better results in all parameters. One explanation might be the much wider spread of the outcome results (range) in the failure group and the limited number of patients in general. Another explanation might be the tear configuration—including chronic tears—and different tear configurations—including rarely massive tears or an intact force couple.

The literature on outcomes after successful and failed RC repair is conflicting.^23,31,36,42,50,52,56^ Similar to the presented results, a study of 1600 consecutive patients with RC repair found no significant difference between the successful and failed repairs.^49^ Those results were also confirmed in a recent systematic review that successful repair was not superior to failed repair in 46 out of 72 studies whereas successful repair showed better results in 17 of 72 studies.^39^ However, other systematic reviews^23,52^ have found significantly better clinical outcome scores for successful RC repairs compared with failed RC repairs. One very recent systematic review^23^ described improvements in clinical outcomes after successful RC repair. However, the mean difference measured 6 points for the CS, which might not be clinically relevant. According to Jost et al,^27^“The potential for rerupture should not be considered a formal contradiction to an attempt at repair if optimal function recovery is the goal of treatment”. Interestingly, most studies focused on short-term data. Some recently published articles^40,56^ showed specifically better results after 10 years of follow-up for successful compared with failed RC repairs.

The authors appreciate the limitations of the study. First, only a part of the complete cohort of patients was analyzed, which resulted in smaller case numbers than possible. However, no previous study has reported outcomes at the 5-year minimum follow-up. Second, a certain number of patients needed to be reported as lost to follow-up. Third, the segmentation method of the supraspinatus muscle was done by 2 examiners manually with individual decisions on whether to define certain parts of the MRI slices as muscle or not. This could be achieved automatically in future applications using artificial intelligence or cluster learning with potentially more accurate results. Fourth, acute and chronic tears were included in this study, which might be conflicting for the outcome analysis. Fifth, the study design was possibly underpowered for some outcome parameters. Especially, clinical outcome scores reached a power of 0.6 in the post hoc power analysis for independent pairs. To account for that, we matched the patients, which resulted in improved power. Unfortunately, the number of included patients was defined by the number of failed repairs. A larger patient cohort or a slightly different study design would be necessary to explore those outcome parameters further. Sixth, for calculation of fatty infiltration at the last follow-up of at least 60 months, a correction factor had to be applied due to changes in the MRI protocols over the years.

## Conclusion

Successful RC repair was associated with a significant increase in 3D supraspinatus muscle volume and halt of 3D fatty infiltration at a midterm follow-up of 5 years. However, failed RC repair also showed improvement of volume, but increase of fat fatty infiltration.

## Supplemental Material

sj-pdf-1-ajs-10.1177_03635465241299795 – Supplemental material for 3D Muscle Volume and 3D Fat Fraction After Successful and Failed Arthroscopic Rotator Cuff Repair at 5-Year Follow-up Supplemental material, sj-pdf-1-ajs-10.1177_03635465241299795 for 3D Muscle Volume and 3D Fat Fraction After Successful and Failed Arthroscopic Rotator Cuff Repair at 5-Year Follow-up by Philipp Kriechling, Jethin Joshy, Stefan Klotz, Georg C. Feuerriegel, Philipp Fürnstahl, Reto Sutter, Mazda Farshad and Karl Wieser in The American Journal of Sports Medicine

## Footnotes

¶ References: 2, 3, 7, 9, 11, 13, 15, 16, 18-22, 25, 30, 35, 43-45, 54, 57, 61.
