# Supplementary material for: 3D Muscle Volume and 3D Fat Fraction After Successful and Failed Arthroscopic Rotator Cuff Repair at 5-Year Follow-up
Source: Am J Sports Med. 2025 Mar 1;53(3):571–82. doi: 10.1177/03635465241299795 (PMC11874612; doi:10.1177/03635465241299795)
Supplement: sj-pdf-1-ajs-10.1177_03635465241299795 – Supplemental material for 3D Muscle Volume and 3D Fat Fraction After Successful and Failed Arthroscopic Rotator Cuff Repair at 5-Year Follow-up [file sj-pdf-1-ajs-10.1177_03635465241299795.pdf]

Improvement of 3D Muscle Volume and 3D Fat Fraction after Successful and Failed Arthroscopic Rotator Cuff Repair after 5-Year Follow-up

APPENDIX

Table A1: Change in muscle volume and fat fraction

Mean Change In Supraspinatus Muscle Volume & Fat Fraction Preoperative, 3-, 12- and 60-months Postoperative & P-Values[1]

	Intact (n =21)					Failure (n = 18)					P-Value[1]
	Mean	SD	Median	Max	Min	Mean	SD	Median	Max	Min	
**SSP[2] Volume[3]**											
Preop – 3 months Postop	-1.20	4.11	-1.68	7.65	-9.72	-3.07	3.66	-2.83	3.32	-11.11	.158
Preop – 12 months Postop	1.22	3.89	.42	9.37	-6.16	-2.49	5.07	-1.55	6.94	-12.66	**.015**
Preop – 60 months[4] Postop	6.58	4.42	6.06	16.89	-.43	1.18	5.97	-.33	14.32	-8.45	**.006**
3 months Postop – 12 months Postop	2.30	3.26	2.23	10.04	-2.06	.58	3.55	.18	7.12	-6.94	.149
3 months Postop – 60 months[4] Postop	7.75	4.80	7.59	17.56	-3.90	4.25	4.55	2.97	11.97	-1.20	**.022**
12 months Postop – 60 months[4] Postop	5.35	3.59	5.28	13.43	-3.92	3.67	3.25	4.56	9.69	-1.64	.126
**SSP[2] 2D Fat Fraction**											
Preop – 3 months Postop	0.3%	2.8%	1%	5%	-8%	3%	5.7%	3%	18%	-6%	.061
Preop – 12 months Postop	-0.2%	3.4%	0%	9%	-5%	3.3%	5.9%	3%	13%	-8%	**.028**
Preop – 60 months[4] Postop	0.02%	5.5%	-0.7%	19.1%	-7.8%	8.6%	9.1%	6.7%	29%	-6.8%	**<.001**
3 months Postop – 12 months Postop	-0.3%	3.2%	0%	9%	-6%	0.9%	4.4%	3%	6%	-9%	.129
3 months Postop – 60 months[4] Postop	-0.01%	5.3%	-0.4%	19.1%	-5.6%	5.3%	9.0%	2.4%	22.4%	-7.8%	**.044**
12 months Postop – 60 months[4] Postop	0.25%	3.0%	-0.2%	10.1%	-3.2%	5.2%	7.6%	2.7%	19.8%	-6.0%	**.018**
**SSP[2] 3D Fat Fraction**											
Preop – 3 months Postop	1.1%	1.1%	0.9%	3.6%	-0.6%	2.8%	2.9%	2.8%	10.7%	-0.7%	**0.046**
Preop – 12 months Postop	0.8%	2.2%	0.9%	5.1%	-3.7%	2.8%	2.7%	3.1%	6.2%	-2.9%	**0.01**
Preop – 60 months[3] Postop	0.6%	2.7%	1.0%	7.4%	-4.8%	6.8%	6.2%	7.0%	19.0%	-1.4%	**.001**
3 months Postop – 12 months Postop	-0.2%	1.9%	-0.1%	3.7%	-6%	-1.3%	7.2%	1%	3.1%	-26.4%	0.313
3 months Postop – 60 months[3] Postop	-0.2%	2.5%	-0.3%	6.0%	-4.9%	2.3%	8.8%	1.6%	14.3%	-24.7%	**.024**
12 months Postop – 60 months[3] Postop	0.1%	1.5%	0.1%	2.6%	-3.8%	3.9%	5.3%	1.6%	13.4%	-2.3%	**.026**

[1] Mann-Whitney Test

[2] SSP = Supraspinatus

[3] Volume measured in cm^3

[4] At least 60 months Postoperative

Table A2: P-Values for change in muscle volume and fat fraction

	P-Values[1] of Supraspinatus Muscle Volume & Fat Fraction Preop, 3-, 12- and 60-months Postop	
	Intact (n = 21)	Failure (n = 18)
**SSP[2] Volume**		
Preop – 3 months Postop	.135	**.004**
Preop – 12 months Postop	.192	**.048**
Preop – 60 months[3] Postop	**<.001**	.586
3 months Postop – 12 months Postop	**.006**	.446
3 months Postop – 60 months[3] Postop	**<.001**	**.001**
12 months Postop – 60 months[3] Postop	**<.001**	**.002**
**SSP[2] 2D Fat Fraction**		
Preop – 3 months Postop	.406	**.041**
Preop – 12 months Postop	.659	**.032**
Preop – 60 months[3] Postop	.376	**.001**
3 months Postop – 12 months Postop	.467	.319
3 months Postop – 60 months[3] Postop	.316	**.042**
12 months Postop – 60 months[3] Postop	.896	**.012**
**SSP[2] 3D Fat Fraction**		
Preop – 3 months Postop	**.001**	**.003**
Preop – 12 months Postop	.0.91	**.002**
Preop – 60 months[3] Postop	.313	**.001**
3 months Postop – 12 months Postop	.913	.408
3months Postop – 60 months[3] Postop	.778	**.065**
12 months Postop – 60 months[3] Postop	.756	**.0.009**

[1] Wilcoxon Signed Ranks Test

[2] SSP = Supraspinatus

[3] At least 60 months Postop

Table A3: Mean Change Clinical parameters

	Mean Change Absolute and Relative Constant Score and Subjective Shoulder Value Preop, 3-, 12- and 60-months Postop & P-Values[1]										
	Intact (n =21)					Failure (n = 19)					P-Value[1]
	Mean	SD	Median	Max	Min	Mean	SD	Median	Max	Min	
**Absolute Constant Score**											
Preop – 3 months Postop	-5.71	15.20	-11.00	28.00	-28.00	-5.50	16.89	-8.50	27.00	-32.00	.945
Preop – 12 months Postop	16.10	18.68	10.00	61.00	-4.00	14.50	21.21	8.50	66.00	-25.00	.813
Preop – 60 months2 Postop	18.52	16.67	14.00	60.00	-3.00	13.17	21.91	6.50	64.00	-23.00	.183
3 months Postop – 12 months Postop	21.81	9.26	24.00	41.00	5.00	20.00	8.25	18.00	40.00	4.00	.549
3 months Postop – 60 months[2] Postop	24.24	9.32	25.00	40.00	8.00	18.67	11.36	15.50	45.00	3.00	.065
12 months Postop – 60 months[2] Postop	2.43	6.21	1.00	16.00	-9.00	-1.33	6.65	-2.00	14.00	-15.00	.126
**Relative Constant Score**											
Preop – 3 months Postop	-6.82	15.96	-10.90	28.00	-29.19	-6.52	17.41	-9.91	27.00	-33.50	.900
Preop – 12 months Postop	16.92	19.53	11.10	64.87	-3.60	15.41	22.03	9.39	67.87	-26.89	.900
Preop – 60 months[2] Postop	21.21	17.04	17.58	64.04	.10	15.73	22.14	9.51	66.04	-22.89	.192
3 months Postop – 12 months Postop	23.74	9.98	25.60	43.21	5.40	21.92	8.40	21.14	41.87	4.00	.568
3 months Postop – 60 months[2] Postop	28.03	9.65	28.55	44.75	11.88	22.25	11.37	19.68	47.38	4.00	.073
12 months Postop – 60 months[2] Postop	4.29	7.19	3.70	20.70	-9.17	.33	6.86	.28	14.58	-16.75	.119
**Subjective Shoulder Value**											
Preop – 3 months Postop	-.43	28.69	5.00	40.00	-60.00	11.67	34.81	15.00	75.00	-65.00	.337
Preop – 12 months Postop	31.71	21.58	35.00	60.00	-10.00	26.56	32.31	30.00	89.00	-40.00	.432
Preop – 60 months[2] Postop	35.40	18.85	40.00	65.00	.00	30.44	35.48	34.00	90.00	-60.00	.561
3 months Postop – 12 months Postop	32.14	17.90	30.00	70.00	.00	14.89	15.46	12.00	40.00	-20.00	**.005**
3 months Postop – 60 months[2] Postop	35.83	20.03	35.00	75.00	.00	18.78	16.94	16.50	50.00	-10.00	**.008**
12 months Postop – 60 months[2] Postop	3.69	8.25	2.50	25.00	-10.00	3.89	12.56	.50	30.00	-20.00	.900

[1] Mann-Whitney Test

[2] At least 60 months Postop

Table A4: P-Values for mean clinical change

	P-Values[1] Absolute and Relative Constant Score, Subjective Shoulder Score Preop, 3-, 12- and 60-months Postop	
	Intact (n = 21)	Failure (n = 18)
**Absolute Constant Score**		
Preop – 3 months Postop	.099	.209
Preop – 12 months Postop	**<.001**	**.003**
Preop – 60 months[2] Postop	**<.001**	**.013**
3 months Postop – 12 months Postop	**<.001**	**<.001**
3 months Postop – 60 months[2] Postop	**<.001**	**<.001**
12 months Postop – 60 months[2] Postop	.165	.365
**Relative Constant Score**		
Preop – 3 months Postop	.063	.133
Preop – 12 months Postop	**<.001**	**.004**
Preop – 60 months[2] Postop	**<.001**	**.003**
3 months Postop – 12 months Postop	**<.001**	**<.001**
3 months Postop – 60 months[2] Postop	**<.001**	**<.001**
12 months Postop – 60 months[2] Postop	**.014**	.670
**Subjective Shoulder Value**		
Preop – 3 months Postop	.872	.169
Preop – 12 months Postop	**<.001**	**.010**
Preop – 60 months[2] Postop	**<.001**	**.007**
3 months Postop – 12 months Postop	**<.001**	**.003**
3 months Postop – 60 months[2] Postop	**<.001**	**<.001**
12 months Postop – 60 months[2] Postop	.060	.180

[1] Wilcoxon Signed Ranks Test

[2] At least 60 months Postop

Table A5: Sugaya rating per ID

Sugaya values preop, at 3-, 12- and 60-months Postop[1] in all patients

	Group	3 months postop	12 months postop	60 months postop
Patient ID's				
1	Intact	2	2	2
9	Failure	5	5	5
12	Intact	2	2	2
14	Failure	5	5	5
16	Intact	2	2	2
22	Intact	2	2	2
24	Intact	2	2	2
32	Failure	5	5	5
36	Failure	5	5	5
38	Intact	2	1	2
42	Failure	4	4	4
44	Intact	2	2	1
46	Failure	4	4	4
48	Failure	4	4	5
49	Intact	2	2	2
51	Intact	2	1	1
53	Intact	2	2	1
56	Failure	2	5	5
62	Intact	2	2	1
67	Failure	4	4	4
68	Intact	2	1	1
69	Failure	3	4	4
72	Failure	4	4	4
73	Intact	2	1	1
74	Intact	2	1	2
76	Intact	3	2	2
77	Intact	2	2	2
81	Intact	2	1	1
83	Intact	2	2	2
85	Failure	4	4	4
87	Failure	2	4	4
89	Intact	2	2	2
90	Intact	2	2	1
91	Failure	4	4	4
92	Intact	2	2	1
93	Failure	5	5	5
95	Failure	5	5	5
104	Failure	5	5	5
107	Failure	5	5	5

[1]At least 60 months Postop

Table A6: Sugaya rating change over time

TABLE 5
Sugaya Values 12- and 60-months[1] Postoperative with Intergroup Comparison

Group	Muscle	Sugaya	12 months	60 months	P-Value[2]
Intact	SSP	Sugaya I	n = 6	n = 9	0.34
		Sugaya II	n = 15	n = 12	
		Sugaya III	n = 0	n = 0	
		Sugaya IV	n = 0	n = 0	
		Sugaya V	n = 0	n = 0	
Failure	SSP	Sugaya I	n = 0	n = 0	
		Sugaya II	n = 0	n = 0	
		Sugaya III	n = 0	n = 0	
		Sugaya IV	n = 9	n = 8	
		Sugaya V	n = 9	n = 10	
Intact	ISP	Sugaya I	n = 4	n = 0	0.15
		Sugaya II	n = 4	n = 4	
		Sugaya III	n = 0	n = 0	
		Sugaya IV	n = 0	n = 2	
		Sugaya V	n = 0	n = 1	
Failure	ISP	Sugaya I	n = 13	n = 6	
		Sugaya II	n = 1	n = 1	
		Sugaya III	n = 0	n = 0	
		Sugaya IV	n = 0	n = 2	
		Sugaya V	n = 0	n = 0	
Intact	SSC	Sugaya I	n = 6	n = 15	0.55
		Sugaya II	n = 9	n = 2	
		Sugaya III	n = 0	n = 0	
		Sugaya IV	n = 0	n = 0	
		Sugaya V	n = 0	n = 0	
Failure	SSC	Sugaya I	n = 1	n = 12	
		Sugaya II	n = 8	n = 1	
		Sugaya III	n = 0	n = 0	
		Sugaya IV	n = 1	n = 4	
		Sugaya V	n = 0	n = 0	
